# Mental Health in the Chilean Incarcerated Population: A Screening Approach

**DOI:** 10.3390/ijerph22020268

**Published:** 2025-02-12

**Authors:** Guillermo Sanhueza, Jessica Candia, Liza Zúñiga

**Affiliations:** 1 School of Social Work, Loyola University Chicago, Chicago, IL 60611, USA; 2 Vicerrectoría de Investigación e Innovación, Universidad Arturo Prat, Iquique 1110011, Chile; 3 School of Social Work, University of the Americas, Campus Talcahuano, Concepción 4100000, Chile; jessica.candia@gmail.com; 4 Interdisciplinary Doctoral Program in Social Sciences, Faculty of Social Sciences, Campus Juan Gómez Millas, University of Chile, Santiago 7800284, Chile; liza.zuniga.c@gmail.com

**Keywords:** mental health, incarcerated persons, prisons, Chile, GHQ-12 instrument

## Abstract

From both a social and epidemiological perspective, incarcerated individuals in Latin America face a series of accumulated disadvantages in different areas, one of them being the deterioration in their mental health linked to confinement and the negative effects of incarceration. However, since mental health evaluations at the intake phase are virtually non-existent for incarcerated populations in Chile, the diagnosis of, monitoring of, and intervention initiatives on mental health issues are very limited, thus limiting the possibilities of causal relationship analysis and evidence-based interventions. Thus, the first step to making the topic of mental health more visible to public policy is to gather more evidence about it in prison settings. This article analyzed—using a screening approach—the presence, suspicion, or absence of psychopathology using the GHQ-12 instrument among a sample of 1159 incarcerated individuals grouped in 20 prisons throughout Chile. Our main results show that there are about 22.3% of Chilean inmates with presence of some form of psychopathology, plus an additional 51.8% with the criteria for suspected psychopathology; we also found significant, bivariate relationships between various mental health items and gender, the type of prison, and age, so that incarcerated women, younger inmates, and those housed in public prisons reporting more problems than their counterparts. Finally, we discuss the implications of our findings for the improvement of prison life in Chile and the possibility of social reintegration for incarcerated people.

## 1. Introduction

Prisons in Chile currently house more than 60,000 people, distributed throughout the country in 79 facilities, which, altogether, have an occupancy rate of over 130% [1]. Although the prison system in Chile is less precarious than in other countries in the region [2], when compared to developed countries, Chilean prisons are far from achieving social reintegration and reducing recidivism. Indeed, the problems of overcrowding and insufficient infrastructure; high levels of violence among inmates; institutional abuse; limited access to reintegration programs; various legal barriers once the person leaves prison; corruption within penitentiary facilities; and poorly trained staff are characteristics of prisons in Chile [2,3,4,5,6].

Populations in contexts of confinement are a social group that faces a high prevalence of mental disorders [7] that are activated, aggravated, or generated as a response to incarceration and forced coexistence [8,9]. Now, if confinement is problematic, it is even more so in the precariousness of prison conditions in Latin America, marked by historical abandonment and the feeling of permanent crisis expressed in overcrowding and overpopulation, deteriorated infrastructure, violence among inmates themselves, or institutional abuse [10]. Added to the above is the scarce or non-existent psychological care that is provided during incarceration [3], making the discussion of mental health in contexts of confinement paradoxical from the very beginning [11].

Aligned with the regional situation, the mental health of persons deprived of liberty (PDLs) in Chile is a current problem, but it has recently worsened amid growing overcrowding and increased violence within the Chilean penitentiary system [2]. Previous studies suggested that the prison population would have a greater vulnerability than the general population [12] with respect to depressive disorders, anxiety, psychotic disorders, substance or alcohol abuse [13], and greater suicide risk [14], and they also point out that these problems would have been dragging on since adolescence [15]. Other studies have emphasized that the rates of mental health problems in the Chilean deprived population would be lower than in other countries with middle and low incomes [7].

Other studies have indicated that the prevalence of mental illness in the prison population in Chile was around 14% [16]; León-Mayer and his colleagues [17] identified a prevalence of antisocial personality disorder in just over two-thirds and estimated that just over half (56.5%) had problematic or abusive patterns of alcohol consumption. Regarding drug use, 24.4% consumed only one type of drug and 30.1% consumed two types of drugs; 40.7% of inmates reported that they had been diagnosed with a learning disorder or hyperactivity during their childhood [18]. Data provided by the National Health Plan [19] mentioned that PDLs had a prevalence of mental disorders of 34.1% (accused) and 23.6% (convicted), albeit with data from 2007.

In the case of incarcerated women, who had a high deterioration in their mental health from before incarceration, there was not much in the way of data available. A couple of exceptions were two studies carried out in Chile in 2018, suggesting traumatic situations of violence, mistreatment, and abuse that many of them had experienced in their childhood and adolescence and that are linked, in turn, to serious mental health problems [4]. A report by the Subcommittee for the Prevention of Torture reported mental health problems for several inmates in the prisons of Antofagasta and Valdivia, although without offering systematic data for this segment at the national level [20].

Thus, one of the knowledge gaps when talking about mental health in contexts of confinement in Latin America, especially after the COVID-19 pandemic, is to update empirical evidence to guide interventions with incarcerated people. In the specific case of Chile, it is also important to analyze the mental health situation of PDLs due to the important legal gaps in this matter [21], including the absence of a criminal enforcement law in the country that could better regulate the deprivation of liberty [5].

Thus, to help close part of this gap, and based on data from a national study carried out between 2022 and 2023, this article seeks to describe the mental health situation—from a screening approach—in a random sample of incarcerated individuals from 20 Chilean prisons, identifying different profiles in terms of the presence, suspicion, or absence of psychopathology. We believe that this approach is novel in methodological terms and contributes to the identification of needs and the future implementation of mental health interventions for PDLs, reducing the dehumanization and inequity that affect this sector of the population [22].

## 2. Materials and Methods

This study was conducted in the context of a larger, publicly funded collaborative research-and-development (R&D) project between the [anonymized] University and the Chilean prison service (Gendarmería de Chile), implemented in the 16 regions of the country between 2022 and 2023. For this specific study, we answered our research objective using the General Health Questionnaire GHQ-12 instrument, applied to a two-stage sampling procedure: first, we purposively selected a sample of 20 prison facilities, considering the largest, most criminologically diverse prison of each region of the country as the main selection criterion; secondly, we applied a simple, random sampling strategy to select individuals within each predetermined facility, with a 95% confidence interval. We only considered already-sentenced individuals. Each pre-selected inmate was asked about their voluntary participation in the study through informed consent. Once this was ratified, each person completed the Measuring Quality of Prison Life [MQPL] questionnaire and the General Health Questionnaire [GHQ-12] in 30–40 min. In some cases, when an individual had reading or writing issues, this person was assisted by one trained interviewer. No monetary incentives were given for participation, and no replacements were chosen. The study was approved by the Ethics Committee of [anonymized]. Due to considerations of anonymity and confidentiality, we were unable to report any particular case to the prison administration; instead, we prepared a thorough report for the prison service with aggregated data per facility, including recommendations for a further, in-depth evaluation.

Our study focused on prison facilities, excluding other forms of progressive sentences. The sampling mechanism included a two-stage process: firstly, a purposive sample was compiled, considering the representation of every region of the country but prioritizing the biggest facilities, with at least one facility per region (usually located in regional capitals) where the highest number of penal populations are concentrated in prisons of major complexity, with the highest levels of overpopulation and recidivism. In the case of the Metropolitan region (the capital of the country), the survey was conducted in 5 prisons, considering that almost half of the national population lives in the capital city. Although this type of sampling does not statistically represent the total penal population, it has the advantages of being time-efficient, covering different regions of the country, and focusing on more complex, populated prisons. The details about sample sizes and response rates by facility can be found in Table 1.

Data collection was carried out in various physical spaces in the selected prisons, including classrooms, libraries, gymnasiums, chapels, and multipurpose rooms. Although the study included the collaboration of prison staff, during the application of the instrument, we took the precaution to ensure that prison officers were not present in the same physical space, in order to reduce social desirability or evaluative apprehension. The average response rate reached 81.5%.

The psychological distress screening questionnaire (GHQ-12) was initially developed by Goldberg and Williams [23] and is one of the most widely used instruments in the world due to its good psychometric properties—with good levels of internal consistency and the capability of detecting symptoms of common mental disorders of a depressive or anxious nature—in community settings or in non-psychiatric clinical settings [24,25]; it is self-administered and consists of 12 items (6 positive and 6 negative) and has been validated in various population groups [26,27,28], including penal populations. Its sensitivity (0.70) and specificity (0.80) against standardized psychiatric interviews are acceptably high [29].

The questionnaire inquires about loss of concentration, loss of sleep, useful performance in life, decision making, feeling overwhelmed and tense, overcoming difficulties, enjoyment of daily activities, coping with problems, feelings of depression, loss of self-confidence, doubt about the value of life, and feelings of happiness. The GHQ-12 assesses a general dimension of self-perceived health and also allows for the distinction of two subdimensions: psychological well-being, with its items 1, 2, 4, 7, 10, and 12, and a subdimension of social functioning and coping, with its items 3, 5, 6, 8, 9, and 11. To determine the absence, suspicion, or presence of psychopathology with GHQ-12 in this study, the “GHQ-12 scoring method” was used, where the Likert scale score (0 to 3) is transformed into a dichotomous score (0 to 1), obtaining a score between 0 and 12 [28]. This questionnaire has been previously tested in prison populations elsewhere [29,30,31], and its sensitivity and specificity are acceptably high.

It is worth highlighting that the sensitivity and specificity depend on the cutoff points used; the punctuations usually used are binary scales (0-0-1-1) and Likert scales of 4 points (0-1-2-3), where the highest punctuations indicate a considerable worsening. A punctuation above a specific cutoff (3–4 for bimodal and 13/14 for the Likert scale) indicates psychological discomfort and suggests more inquiry for possible mental disorders [32]. Nonetheless, the same cutoffs have been previously used in the penal population. A recent study in Israel rated the items on a Likert scale ranging from 0—‘not at all’—to 3—‘much more than usual’. A score of 1 was assigned to the responses ‘rather more’ or ‘much more than usual’ and 0 to ‘not at all’ or ‘no more than usual’. The final score was computed as the sum of all items (range 0 to 12). Scores of four or more were set to indicate GHQ ‘caseness’, indicating a possibility for a common mental health disorder CMD and a need for assessment and treatment [30]. Higher cutoffs have been used in specific settings [33,34,35], and the thresholds apply to the most commonly used scoring methods, not the Likert or chronic scales. The disagreement between different studies highlights a debate about GHQ and how to select appropriate thresholds for the sample of interest. The recommendation from the scale’s author is to carry out a validation study in the population of interest [36], but the associated expense and impracticality are often a deterrent. Thus, in 1998, the GHQ’s author and others recommended an alternative to a validation study: the mean score method to set a crude threshold [37]. Within prison populations, there is evidence that other instruments may be more sensitive, but this varies according to context [28].

## 3. Results

In terms of sociodemographic characteristics, our final sample consisted of 1159 surveys, of which 94% of respondents were men and 6% were women. The average sample age was 37.7 years, and 34.7% of respondents reported having gone through state-protective services during infancy or adolescence—Sename. In addition, 94.4% were Chilean nationals and more than half of the respondents had been incarcerated before (56.2%). Regarding formal schooling, 55.9% had completed primary education (equivalent to kindergarten through eighth grade), 19.6% had completed secondary education (equivalent to ninth grade through twelfth grade), and only 3.2% had completed higher education. Almost two-thirds of the sample (65.8%) reported having been incarcerated in that same prison for more than two years, and nearly 58% have sentences until 2025 or longer.

In terms of prison conditions, and based on our survey results, respondents indicated that the most problematic deficits were the inadequate temperatures in cells or modules (average of 2.67 measured on a scale of 1–5); the scarce access to reintegration programs, especially the difficulty of accessing paid work (average 2.32); and difficulties in accessing psychological care in prisons (average 2.40).

When asked about interactions with other inmates (Cronbach’s Alpha = 0.74), the most negative results reported were “in this prison, the weakest inmates were abused” (3.25, on a scale of 1–5, where 1 is never and 5 is always) and “in this prison I fear for my physical integrity” (2.62). Regarding the inmate–officer relationship (Cronbach’s Alpha = 0.92), the lowest items were “I feel that the officers trust me” (2.77) and “the gendarmes [officers] care about me” (2.84).

Considering the prison regime (Cronbach’s Alpha = 0.80), the scores were inmates’ lack of knowledge regarding how to make formal complaints (2.88), and the long response times from the administration (2.90). Regarding the operation of the prison (Cronbach’s Alpha = 0.83), the main problems detected were boredom (“my life in this prison is boring” = 3.72), the tense prison environment (“the atmosphere of this prison is tense” = 3.45), and the perception of corruption (“in this prison things are moved with money” = 3.40). Likewise, the report on drug use was high (“there is a lot of drug use in this prison” = 3.18).

In terms of the screening of psychopathology in persons deprived of liberty, we employed the “GHQ scoring method”, finding that 22.3% of the sample showed signs of the presence of psychopathology. Likewise, when analyzed by area, the southern part of the country presents a higher percentage of people with signs of the presence of psychopathology, at 24.5%, compared to the northern and central areas (Table 2).

The total percentage of persons deprived of liberty in the sample with the presence of some psychopathology (22.3%), although seeming to be higher than the general population [12], is lower in comparison with other prison populations in the region, including Colombia, where 43.7% of persons deprived of liberty had traits of depression and 85.7% had traits of anxiety [38]; Spain, where at least one mental disorder in the last month was found in 25.8% of the male population [39]; Holland, where the prevalence of common mental disorders such as anxiety, depression, and somatoform disorders was as high as 62.7% [40]; or the United Kingdom, where the prevalence of mental disorders was up to 15 times higher in persons deprived of liberty than in the general population [41].

We also conducted a series of bivariate association tests to examine whether items on the GHQ-12 varied, according to gender (male or female), type of prison (public or private), and age. Our results showed that, in terms of gender differences, women exhibited significant differences on items g1, g2, g4, and g6. Thus, women reported more trouble sleeping (“My worries have made me lose sleep”; t = 2.13; *p* = 0.016), a higher perception of distress (“I have felt constantly overwhelmed or under great stress”; t = 3.27; *p* = 0.000), experiencing more sadness (“I have felt unhappy or depressed”; t = 2.45; *p* = 0.007), and feeling more useless than men (“I have thought that I am a good-for-nothing person”; t = 1.89; *p* = 0.029). Detailed results for significant differences only between men and women are shown in Table 3.

When the type of prison was considered, inmates in public facilities reported higher values for items g1, g2, g3, and g4 and lower for g10. In other words, incarcerated individuals in public facilities reported being more worried (“my worries have made me lose sleep”; t = 2.38, *p* = 0.008), a higher perception of distress (“I have felt constantly overwhelmed or under great stress”; t = 3.97, *p* = 0.000), a greater perception of being unable to overcome difficulties (“I have had the feeling that I cannot overcome my difficulties”; t = 2.96, *p* = 0.001), and more sadness (“I have felt unhappy or depressed”, t = 4.32, *p* = 0.000) than inmates inhabiting private facilities. Individuals in private prisons reported enjoying more their daily activities than their counterparts in public facilities (“I have been able to enjoy my daily activities here in prison”; t=1.898, *p* = 0.029). The detailed results for the comparison between public and private prisons are shown in Table 4.

When age and scores of mental health were analyzed, age had a positive (yet small) statistical association with g8 and g10 and showed a negative (but small) significant correlation with items g2, g4, g5, and g9. This means that younger inmates experienced more difficulties in terms of feeling overwhelmed (r = −0.078) and sad or depressed (r = −0.108), with less confidence in themselves (r = −0.075); at the same time, younger inmates felt better at making decisions (r = −0.062). On the other hand, older inmates reported more feelings of playing an important role in life (r = +0.101) and were more able to enjoy daily life activities in prison (r = +0.062) in comparison to younger inmates.

As Table 5 shows, the most worrying average values appear for the items “my worries have made me lose sleep” (1.45 on a 0–3 scale) and “I have constantly felt overwhelmed or under great tension” (1.33 on a 0–3 scale), which is consistent with the experience of deprivation of liberty in the midst of precarious contexts, marked by overcrowding and violence.

As shown in Table 6, the most prevalent affected areas in PDLs correspond to feelings of being unhappy or depressed (25.7%), worries that have made them lose sleep (24.1%), and feelings of not having an important role in life (18.7%), which is understandable due to the situation of confinement in which they find themselves. Another analysis shows that 26.7% of prisoners have 5–6 aspects of social functioning and coping negatively affected, and 10.6% have 5–6 aspects affected in psychological well-being.

## 4. Conclusions and Discussion

Despite the international obligations to protect the mental health of persons deprived of liberty within the international framework of Human Rights [42], in Latin America, there are problems related to the precarious conditions of confinement, where overcrowding and unsanitary conditions in cells, food, and sanitary services are often the constant in a prison system in permanent crisis [3,10].

At the same time, while incarcerated individuals in Latin America and Chile are suspected to have mental health problems, there are important gaps in the empirical evidence available at the national level for this segment of the population, including a lack of assessment of mental health at intake. In this regard, we believe that one of the main contributions of our study is making more visible the situation of the mental health of the incarcerated. Our results suggest that 22.3% of the sample reported the presence of some psychopathology, a figure higher than the percentage for the general Chilean population.

Variations by gender revealed that women seemed to have more feelings of depression, sadness, and feeling useless than men. These results are consistent with Chilean prisons lacking a well-implemented, gender-sensitive perspective, combined with a patriarchal society that doubly punishes incarcerated women as “bad mothers” [4]. In addition, inmates in public prisons—prisons that are usually older and more deteriorated in terms of infrastructure conditions—reported more problems in terms of sleeping well, feeling overwhelmed, being unable to overcome difficulties, and feeling sad or depressed than their counterparts in privately operated prisons; all of this is consistent with severe overcrowding, a violent environment, and a significant lack of access to rehabilitative programming [2,3].

However, this study must be interpreted within the framework of some limitations. One of them is related to the partial number of prison facilities visited (only 20 out of 80), which, although important from the point of view of the high number and complexity of the prison population they house, does not necessarily represent all the facilities. Furthermore, our study was unable to identify variations in mental health according to prison stay length, type of crime, or whether inmates were indigenous or not because the original dataset did not incorporate these variables in the first place. In the future, more detailed studies on mental health may address some of these limitations.

Even with its limitations, and in light of the scarce empirical evidence on mental health in Chile and Latin America, this study contributes to filling a gap in terms of providing more initial estimates—and with larger samples of incarcerated people in a significant amount of prison facilities—compared to previous knowledge on the topic. Although we do not provide specific diagnoses, we manage to reasonably estimate problematic mental health situations for each facility visited, opening the door to more detailed studies in terms of diagnosis.

A study conducted in seven prison facilities in Chile by Mundt and colleagues [7] offered prevalence rates for various psychopathologies, including any substance use disorder (12.2%), anxiety disorders (8.3%), affective disorders (8.1%), intermittent explosive disorders (5.7%), ADHD in adults (2.2%), and non-affective psychoses (0.8%). Our estimates, on the other hand, while not allowing specific diagnoses, incorporated 20 prison facilities and offered a screening approach on the percentage of the prison population with the presence (22.3%) or suspicion (51.8%) of a psychopathology of some kind. Both studies also highlight the role of substance use within prisons, which may be seen both as a symptom and as a concurrent factor for the deteriorated mental health of the incarcerated in Chile.

Initiatives aimed at ameliorating the mental health crisis in Chilean prisons should include the prioritization of public facilities, with a special concern for young inmates and incarcerated women. Given the severity of the crisis, additional investments in mental health professionals should be made, but, given the usual scarcity of resources around prisons, a realistic approach should not exclusively rely on “medical” approaches but could also incorporate a variety of initiatives such as group therapeutic workshops, sports-based interventions, and spiritual accompanying, among others, which have proven promising in other prison contexts in Latin America and elsewhere.

At the same time, the vast number of prisons we visited, combined with the random selection of inmates for each of the 20 facilities selected, makes us feel confident that, despite its limitations, our work offers an initial account and additional empirical evidence to reactivate public concerns about the seriousness of the mental health crisis that seems to be incubating in Chilean prisons. This constitutes a normative and social justice imperative [43] to reduce health gaps within prison facilities [44], generate better chances for the social reintegration of incarcerated individuals [45], and create better working conditions for staff [6].

## Figures and Tables

**Table 1:** Prison populations, sample sizes and response rates by facility.

Prison Facility	Prison Population by Center	Expected Sample Size	Effective Sample Size	Response Rate
Arica	1128	93	93	100%
Alto Hospicio	985	75	47	62.6%
Antofagasta	789	65	44	67.6%
Copiapó	265	20	20	100%
La Serena	1726	123	84	68.2%
Valparaíso	1495	106	90	84.9%
Rancagua	1405	104	100	96%
Talca	428	32	32	100%
Chillán	270	20	19	95%
Concepción	334	26	23	88%
Temuco	299	25	10	38%
Valdivia	1006	77	65	85%
Puerto Montt	1179	89	58	65%
Coyhaique	97	8	8	100%
Punta Arenas	251	19	19	100%
Colina I	1869	133	133	100%
Colina II	1196	89	67	74.9%
Puente Alto	682	52	50	96%
Santiago Sur	3565	232	163	70.3%
CPF San Joaquín	446	34	34	100%
Total	19,583	1422	1159	81.5%

**Table 2:** Psychopathology by zone of the country. Sample of incarcerated individuals.

Zone	Frequency	Absence of Psychopathology	Psychopathology Suspected	Presence of Psychopathology
North	235	78 3.2%	111 47.2%	46 19.6%
Center	688	162 23.5%	371 53.9%	155 22.5%
South	184	48 26.1%	91 49.5%	45 24.5%
Totals	1115	288 26%	573 51.8%	246 22.3%

Source: based on data from Project Fondef #id21i10255.

**Table 3:** Statistically significant differences between women and men GHQ-12.

g1. My worries have made me lose sleep	Obs.	Mean	Std. Error	Std. Dev.	95% Confidence Interval	
Women	68	1.720	0.138	1.144	1.443	1.997
Men	1064	1.430	0.033	1.081	1.365	1.495
Combined	1132	1.447	0.032	1.086	1.384	1.511
Difference		0.290	0.135		0.023	0.556
t = 2.137 *p* = 0.016 df = 1130						
g2. I have felt constantly overwhelmed or under great stress						
Women	68	1.735	0.138	1.141	1.459	2.011
Men	1052	1.312	0.031	1.022	1.250	1.374
Combined	1120	1.338	0.030	1.034	1.277	1.399
Difference		0.422	0.128		0.169	0.675
	t = 3.279 *p* = 0.000 df = 1118					
g4. I have felt unhappy or depressed						
Women	69	1.884	0.124	1.036	1.635	2.133
Men	1048	1.582	0.030	0.986	1.522	1.641
Combined	1117	1.600	0.029	0.991	1.542	1.658
Difference		0.301	0.122		0.060	0.543
t = 2.455 *p* = 0.007 df = 1115						
g6. I have thought that I am a good-for-nothing person						
Women	70	0.628	0.138	1.156	0.352	0.904
Men	1049	0.419	0.026	0.872	0.366	0.472
Combined	1119	0.432	0.026	0.893	0.380	0.484
Difference		0.209	0.110		−0.007	0.425
t = 1.898 *p* = 0.029 df = 1117						

**Table 4:** Statistically significant differences on GHQ-12: inmates in public v/s private prisons.

g1. My worries have made me lose sleep	Obs.	Mean	Std. Error	Std. Dev.	95% Confidence Interval	
Public	712	1.507	0.040	1.093	1.426	1.587
Private	420	1.347	0.052	1.069	1.245	1.450
Combined	1132	1.447	0.032	1.086	1.384	1.511
Difference		0.159	0.066		0.028	0.290
t = 2.388 *p* = 0.008 df = 1130						
g2. I have felt constantly overwhelmed or under great stress						
Public	703	1.432	0.039	1.055	1.354	1.510
Private	417	1.179	0.047	0.977	1.085	1.273
Combined	1120	1.338	0.030	1.034	1.277	1.399
Difference		0.252	0.063		0.127	0.377
	t = 3.977 *p* = 0.000 df = 1118					
g3. I have had the feeling that I cannot overcome my difficulties						
Public	707	1.032	0.040	1.067	0.953	1.111
Private	413	0.842	0.047	0.973	0.748	0.936
Combined	1120	0.962	0.031	1.037	0.901	1.023
Difference		0.189	0.064		0.064	0.315
t = 2.966 *p* = 0.001 df = 1118						
g4.I have felt unhappy or depressed…						
Public	703	1.698	0.036	0.978	1.625	1.770
Private	414	1.434	0.048	0.993	1.338	1.530
Combined	1117	1.600	0.029	0.991	1.542	1.658
Difference		0.263	0.060		0.144	0.383
t = 4.324 *p* = 0.000 df = 1115						
g10. I have been able to enjoy my daily activities here in prison						
Public	706	1.644	0.040	1.088	1.564	1.724
Private	414	1.797	0.051	1.056	1.695	1.899
Combined	1120	1.700	0.032	1.079	1.637	1.764
Difference		−0.152	0.066		−0.283	−0.021
t = 1.898 *p* = 0.029 df = 1117						

**Table 5:** Descriptive statistics on GHQ-12 scale applied to incarcerated individuals in Chile.

GHQ-12 Items	*n*	Mean	S.D.	Variance	Asymmetry	Kurtosis
1. My worries have made me lose sleep	1171	1.45	1.08	1.17	0.18	−1.25
2. I have felt constantly overwhelmed or under great stress	1171	1.33	1.03	1.06	0.32	−014
3. I have had the feeling that I cannot overcome my difficulties	1171	0.95	1.03	1.06	0.78	−0.6
4. I have felt unhappy or depressed	1170	1.6	0.98	0.97	0.17	−1.13
5. I have lost confidence in myself	1171	0.59	0.95	0.90	1.49	0.96
6. I have thought that I am a good-for-nothing person	1171	0.43	0.89	0.79	1.96	2.48
7. I have been able to concentrate well on what I do	1171	1.78	1.03	1.06	−0.09	−1.33
8. I have felt that I play a useful/important role in life	1171	1.71	1.14	1.30	−0.15	−1.44
9. I have felt capable of making decisions	1171	2.03	0.97	0.95	−0.36	−1.27
10. I have been able to enjoy my daily activities here in prison	1171	1.70	1.08	1.16	−0.07	−1.34
11. I have been able to cope adequately with my problems	1171	1.84	1	1.01	−0.13	−1.32
12. Considering all my circumstances, I feel reasonably happy	1171	1.67	1.02	1.05	0.08	−1.29

Note: the minimum/maximum ranges from 0 to 3 for each item. It should be noted that there are negative items (1 to 6) and if they are closer to 3, they are understood as a negative situation (discomfort). On the other hand, for positive items (7 to 12), if the average is closer to 3, they are understood as a positive situation (well-being). The rationale for the background color in this table is to emphasize the difference between g1–g6 versus 67-g10: whereas items g1–g6 are negatively stated, items g7–g12 are positively stated

**Table 6:** Item analyses of responses to the GQH-12 scale from incarcerated individuals in Chile.

GHQ-12 Items	No, Never %	The Usual %	Some More Than the Usual %	Way More Than the Usual %
1. My worries have made me lose sleep	22.4	34.7	18.7	24.1
2. I have felt constantly overwhelmed or under great stress	23.4	38.6	19.3	18.7
3. I have had the feeling that I cannot overcome my difficulties	43.2	30.9	13.3	12.5
4. I have felt unhappy or depressed	11.1	43.5	19.7	25.7
5. I have lost confidence in myself	66.2	17.3	8.0	8.4
6. I have thought that I am a good-for-nothing person	77.6	8.7	6.9	6.9
7. I have been able to concentrate well on what I do	9.8	37.2	18	35
8. I have felt that I play a useful/important role in life	18.4	28.9	16.1	36.7
9. I have felt capable of making decisions	4.8	31.9	19.3	44.1
10. I have been able to enjoy my daily activities here in prison	14.5	34.3	17.9	33.3
11. I have been able to cope adequately with my problems	7.7	36.8	19.1	36.5
12. Considering all my circumstances, I feel reasonably happy	10.9	41.9	16.4	30.8

## Data Availability

The original contributions presented in this study are included in the article. Further inquiries can be directed to the corresponding author.

## References

[1] Gendarmería de Chile (2024). Estadísticas de Población Atendida. https://www.gendarmeria.gob.cl/.

[2] Sanhueza G.E., Pérez F., Candia J., Urquieta M.A. (2021). Inmate-on-inmate prison violence in Chile: The importance of the institutional context and proper supervision. J. Interpers. Violence.

[3] Espinoza O., Martínez F., Sanhueza G. (2014). El Sistema penitenciario y su Impacto en los Derechos Humanos de las Personas Privadas de Libertad. Informe sobre Derechos Humanos en Chile 2014.

[4] Sanhueza G.E., Brander F., Reiser L. (2019). Encarcelamiento femenino en Chile. Calidad de vida penitenciaria y necesidades de intervención. Rev. Cienc. Soc..

[5] Castro A., Contreras L., Sanhueza G. (2023). Permisos de salida y libertad condicional como mecanismos de puesta en libertad anticipada en Chile: ¿necesidad de una revisión?. Ius Prax..

[6] Abello C., Pacheco M., Sanhueza G.E. (2023). Funcionarios penitenciarios en Latinoamérica: Calidad de vida, condiciones laborales y principales problemáticas. Rev. Esp. Sanid. Penit..

[7] Mundt A.P., Alvarado R., Fritsch R., Poblete C., Villagra C., Kastner S., Priebe S. (2013). Prevalence Rates of Mental Disorders in Chilean Prisons. PLoS ONE.

[8] López-Marcelino L.M., Saavedra F.J., López A. (2021). Problemas de salud mental en población penitenciaria. Un. Enfoque Salud Pública. Rev. Asoc. Esp. Neuropsiq..

[9] Arroyo-Cobo J.M. (2011). Estrategias asistenciales de los problemas de salud mental en el medio penitenciario, el caso español en el contexto europeo. Rev. Esp. Sanid. Penit..

[10] Fanarraga I., Barthelemy S., Koetzle D., Mellow J. (2022). A content analysis of prison websites: Exploring approaches to rehabilitation in Latin America. Int. J. Offender Ther. Comp. Criminol..

[11] Sanhueza G.E., Candia J. (2019). Acceso a la atención sanitaria en cárceles chilenas: Una mirada desde los internos. Rev. Esp. Sanid. Penit..

[12] Araya R., Rojas G., Fritsch R., Acuña J., Lewis G. (2001). Common mental disorders in Santiago, Chile: Prevalence and socio-demographic correlates. Br. J. Psychiatry.

[13] Aristizábal E.T., Ríos-García A.L., del Pozo-Serrano F.J. (2016). Salud mental, género, educación social en mujeres reclusas del Centro de Rehabilitación Femenino El Buen Pastor de Barranquilla (Colombia). Rev. Salud Uninorte.

[14] Ceballos-Espinoza F., Chávez-Hernández A., Padilla-Gallegos G.M., Leenaars A.A. (2016). Suicidio en las cárceles de Chile durante la década 2006–2015. Rev. Crim..

[15] Maldonado-Fuentes F. (2013). Prevalencia de patologías de salud mental en la población adolescente privada de libertad: Experiencias nacionales y comparadas. Ius Prax..

[16] Botero L., Arboleda G., Gómez A., García M., Agudelo A. (2019). Depresión en personas incluidas en centros penitenciarios: Revisión narrativa. Rev. Fac. Cienc. Salud Univ. Cauca.

[17] León-Mayer E., Cortés M.S., Folino J. (2014). Descripción multidimensional de la población carcelaria chilena. Psicoperspectivas.

[18] Molina-Coloma V., Pérez J.I., Salaberría K. (2022). Perfil sociodemográfico, delictivo y psicopatológico en una muestra de mujeres en prisión. Rev. Iberoam. Diagnóstico Evaluación Psicológica.

[19] Ministerio de Salud, Gobierno de Chile (2017). Plan Nacional de Salud Mental. https://www.minsal.cl/wp-content/uploads/2017/12/PDF-PLAN-NACIONAL-SALUD-MENTAL-2017-A-2025.-7-dic-2017.pdf.

[20] Naciones Unidas Subcomité para la Prevención de la Tortura y Otros Tratos o Penas Crueles, Inhumanos o Degradantes. Visita a Chile del 4 al 13 de Abril de 2016: Observaciones y Recomendaciones Dirigidas al Estado. https://docstore.ohchr.org/SelfServices/FilesHandler.ashx?enc=6QkG1d%2FPPRiCAqhKb7yhsgPEpOPkPvYO%2F7DAnrKRrASeCSZxJynm8Gh12SesHiDLXFrhVtTB66PZWKOGKjnv%2FYyyA5iTQDO%2Bg6KHeTq7EDZcXH2ee4dfwkXhewCfeGhz.

[21] La Rosa V., Martel E. (2020). Salud Mental y Cárceles: Protección al Derecho a la Salud Mental de las Personas Privadas de Libertad en Chile. Master’s Thesis Law Degree.

[22] Reagan L., Kitt-Lewis E., Loeb S.J., Shelton D., Zucker D.M. (2024). Health equity for people living in correctional facilities: Addressing bias, stigma, and dehumanization. Res. Nurs. Health.

[23] Goldberg D., Williams P. (1988). General Health Questionnaire (GHQ).

[24] Burrone M.S., Abeldaño Zuñiga R.A., Susser L., Lucchese M., Enders J.E., Alvarado R., Valencia E., Fernandez A.R. (2015). Evaluación psicométrica y estudio de fiabilidad del cuestionario general de salud (GHQ-12) en consultantes adultos del primer nivel de atención en Córdoba Argentina. Rev. Fac. Cienc. Med. Cordoba.

[25] Urzúa A., Caqueo-Urízar A., Bargsted M., Irarrázaval M. (2015). ¿Afecta la forma de puntuación la estructura factorial del GHQ-12? Estudio exploratorio en estudiantes iberoamericanos. Cad. Saúde Pública.

[26] Paz C., Mascialino G., Adana-Díaz L., Rodríguez-Lorenzana A., Simbana-Rivera K., Gómez-Barreno L., Troya M., Paez M.I., Cárdenas J., Gerstner R.M. (2020). Behavioral and sociodemographic predictors of anxiety and depression in patients under epidemiological surveillance for COVID-19 in Ecuador. PLoS ONE.

[27] Rocha K.B., Pérez K., Rodríguez-Sanz M., Borrell C., Obiols J.E. (2011). Propiedades psicométricas y valores normativos del General Health Questionnaire (GHQ-12) en población general española. Int. J. Clin. Health Psychol..

[28] Dietzel D., Kewley S., McIlroy D., Synnott J. (2022). Prison Mental Health Screening tools: Updated Choices and Sensitivity. Forensic Update.

[29] Aalto A.M., Elovainio M., Kivimäki M., Uutela A., Pirkola S. (2012). The Beck Depression Inventory and General Health Questionnaire as measures of depression in the general population: A validation study using the composite international diagnostic interview as the gold standard. Psychiatry Res..

[30] Nagar M. (2022). Degrees of Freedom: Comparing Mental Distress of Populations with Different Levels of Access to Care-Prisoners, Psychiatric Patients and General Population. Healthcare.

[31] Favril L., van Ginneken E. (2024). Individual and environmental contributors to psychological distress during imprisonment. Eur. J. Criminol..

[32] Goldberg D.P., Gater R., Sartorius N., Ustun T.B., Piccinelli M., Gureje O., Rutter C. (1997). The validity of two versions of the GHQ in the WHO study of mental illness in general health care. Psychol. Med..

[33] Romito P., Saurel-Cubizolles M.J., Lelong N. (1999). What makes new mothers unhappy: Psychological distress one year after birth in Italy and France. Soc. Sci. Med..

[34] Goldberg D.P., Williams P. (1988). The User’s Guide to the General Health Questionnaire.

[35] Shelton N.J., Herrick K.G. (2009). Comparison of scoring methods and thresholds of the General Health Questionnaire-12 with the Edinburgh Postnatal Depression Scale in English women. Public Health.

[36] Goodchild M.E., Duncan-Jones P. (1985). Chronicity and the General Health Questionnaire. Br. J. Psychiatry.

[37] Goldberg D.P., Oldehinkel T., Ormel J. (1998). Why GHQ threshold varies from one place to another. Psychol. Med..

[38] Lopera M., Hernández J. (2020). Situación de salud de la población privada de libertad en Colombia, una revisión sistemática de la literatura. Rev. Gerenc. Políticas Salud.

[39] López M., Saavedra F.J., López A., Laviana M. (2016). Prevalencia de problemas de salud mental en varones que cumplen condena en centros penitenciarios de Andalucía. Rev. Esp. Sanid. Penit..

[40] Adraro W., Kerebih H., Tesema W., Abamecha F., Hailesilassie H. (2019). Nearly three in every five prisoners experience common mental disorders (CMDs) in Jimma correctional institution; south-West Ethiopia. BMC Public Health.

[41] Baranyi G., Scholl C., Fazel S., Patel V., Priebe S., Mundt A.P. (2019). Severe mental illness and substance use disorders in prisoners in low-income and middle-income countries: A systematic review and meta-analysis of prevalence studies. Lancet Glob. Health.

[42] (2008). Reglas de Brasilia Sobre Acceso a la Justicia de las Personas en Condición de Vulnerabilidad. https://www.acnur.org/fileadmin/Documentos/BDL/2009/7037.pdf.

[43] Niño A.C.N., Díaz D.C., Ramírez L.F. (2017). Trastorno mental en el contexto carcelario y penitenciario. Carta Comunitaria.

[44] Vicente B., Saldivia S., Pihán R. (2016). Prevalencias y brechas hoy; salud mental mañana. Acta Bioeth..

[45] Auty K., Liebling A. (2020). Explorando la relación entre el clima social de la prisión y la reincidencia. Justice Q..

